# Biomechanical responses of human lumbar spine and pelvis according to the Roussouly classification

**DOI:** 10.1371/journal.pone.0266954

**Published:** 2022-07-29

**Authors:** Wei Wang, Baoqing Pei, Shuqin Wu, Da Lu, Peiyan He, Chenghao Ma, Xueqing Wu

**Affiliations:** 1 Beijing key Laboratory for Design and Evaluation Technology of Advanced Implantable & Interventional Medical Devices, Beijing Advanced Innovation Center for Biomedical Engineering, School of Biological Science and Medical Engineering, Beihang University, Beijing, China; 2 School of Big Data and Information, Shanxi Polytechnic Institute, Shanxi, China; University of Vigo, SPAIN

## Abstract

**Background:**

Few studies have analyzed the different biomechanical properties of the lumbar with various morphological parameters, which play an important role in injury and degeneration. This study aims to preliminarily investigate biomechanical characteristics of the spine with different sagittal alignment morphotypes by using finite element (FE) simulation and in-vitro testing.

**Methods:**

According to the lumbar-pelvic radiographic parameters of the Chinese population, the parametric FE models (L1-S1-pelvis) of Roussouly’s type (1–4) were validated and developed based on the in-vitro biomechanical testing. A pure moment of 7.5 Nm was applied in the three anatomical planes to simulate the physiological activities of flexion, extension, left-right lateral bending and left-right axial rotation.

**Results:**

The sagittal configuration of four Roussouly’s type models had a strong effect on the biomechanical responses in flexion and extension. The apex of the lumbar lordosis is a critical position where the segment has the lowest range of motion among all the models. In flexion-extension, type 3 and 4 models with a good lordosis shape had a more uniform rotation distribution at each motor function segment, however, type 1 and 2 models with a straighter spine had a larger proportion of rotation at the L5-S1 level. In addition, type 1 and 2 models had higher intradiscal pressures (IDPs) at the L4-5 segment in flexion, while type 4 model had larger matrix and fiber stresses at the L5-S1 segment in extension.

**Conclusion:**

The well-marched lordotic type 3 lumbar had greater stability, however, a straighter spine (type 1 and 2) had poor balance and load-bearing capacity. The hypolordotic type 4 model showed larger annulus fiber stress. Therefore, the sagittal alignment of Roussouly’s type models had different kinetic and biomechanical responses under various loading conditions, leading to different clinical manifestations of the lumbar disease.

## Introduction

The acquisition of an erect position makes humans the only vertebrates to maintain fully upright bipedalism. A series of morphological changes in the spinal anatomy includes the perpendicularization of the pelvis and the development of the sagittal curve of the spine, known as lumbar lordosis and thoracic kyphosis, which are unique to humans. Lumbar lordosis is not found in other species. Although great apes can achieve an upright posture, their entire spine is a large "C", a long kyphosis, unable to sustain a stable upright posture and walk. The S-curve shape of the human spine in the sagittal plane plays a key role in maintaining balance and stability while minimizing the energy consumption of the back musculature [1, 2].

**Fig 1:**
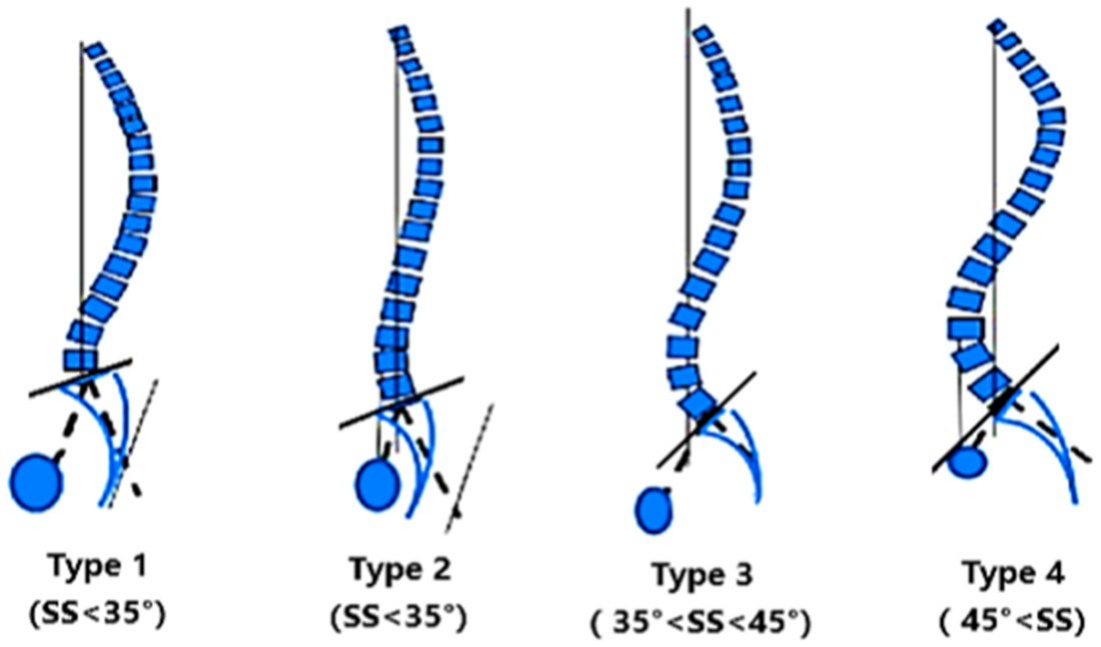
Schematic diagram of Roussouly’s 1–4 morphotypes.

The sagittal alignment of the spine is a recently-developed concept to understand the mechanical equilibrium mechanism and the geometric characteristics of pathological deformity of the spine. It was proposed by Duval-Beaupere et al. [3], who defined pelvic parameters, namely pelvic incidence (PI), sacral inclination (SS), and pelvic tilt (PT). The classification of sagittal alignments has been widely investigated by researchers based on a radiological assessment [4–7]. Four types of sagittal spine alignment were firstly proposed by Roussouly et al. [6] based on the SS and the shape of the spine in 2005 (Fig 1). It is noted that each type of spine has a different pattern of mechanical conduction and balance, which is associated with pathological evolution and postoperative mechanical complications.

Over the past 15 years, epidemiological and clinical studies have demonstrated the importance of sagittal balance in preventing spinal dysfunction and evaluating the outcome of spinal surgery [3, 6, 8, 9]. Clinical studies have hypothesized that the stress distribution of the spine is determined by its structural morphology [2, 10, 11]. For example, a flat or less lordosis spine is subjected to ensure high intervertebral disc pressure, thus having higher occurrences of specific spinal disorders, such as lumbar disc herniation [6, 12, 13]. However, our literature search results showed that the existing studies mainly focus on imaging parameter analysis. There are still little data on the biomechanics of sagittal plane balance. It is probably due to the inherent complexity of mechanical structures of the spine with interacting nonlinear components [14, 15], which makes it impossible to replicate the combined simultaneous effects of body weight and muscle in in-vitro studies. Clinical studies believe that the stress distribution of the spine is determined by its structural morphology. Therefore, a thorough understanding of the mechanical properties of different types of the lumbar-pelvic complex is the key to understanding lumbar degenerative disease and the accelerated degeneration of adjacent segments after fusion.

Therefore, this study aims to preliminarily investigate whether different Roussouly sagittal alignment morphotypes have various kinetic and mechanical characteristics. Based on in vitro biomechanical tests, parametric FE models of Roussouly’s type (1–4) were validated and developed to analyze the biomechanical responses under different loading scenarios, including an intersegment range of motion (ROM), IDP, and maximum stress of the matrix and fibers. These results partially addressed this lack of basic knowledge of the biomechanical characteristics of the spine with various sagittal alignments.

## Methods

### Specimen preparation

Our study have been approved by the biological and medical ethics committee of Beihang University (No:BM20190009). The experimental scheme in this paper meets the ethical requirements and is approved to be implemented. A lumbar (L1–S1, 47.5 years old) was employed from the fresh-frozen human donor spine. To ensure healthy conditions of spine specimen, bony defects, disc degeneration, tumors and scoliosis were excluded from this study. The spiral computed tomography (CT) images with a slice thickness of 0.6 mm (Light Speed Pro16, GE, Waukesha, WI, USA) were obtained to reconstruct the FE model in the next step. The specimen was gradually thawed and carefully dissected to remove the soft tissues, while preserving ligaments, facet joints and the (intervertebral disc) IVDs [16]. The specimen was kept moist with 0.9% saline throughout the testing procedure. The cranial end of vertebra L1 and the caudal end of S1 were embedded in polymethylmethacrylate (PMMA) using custom-made containers for mounting in the testing device.

### Testing protocol

**Fig 2:**
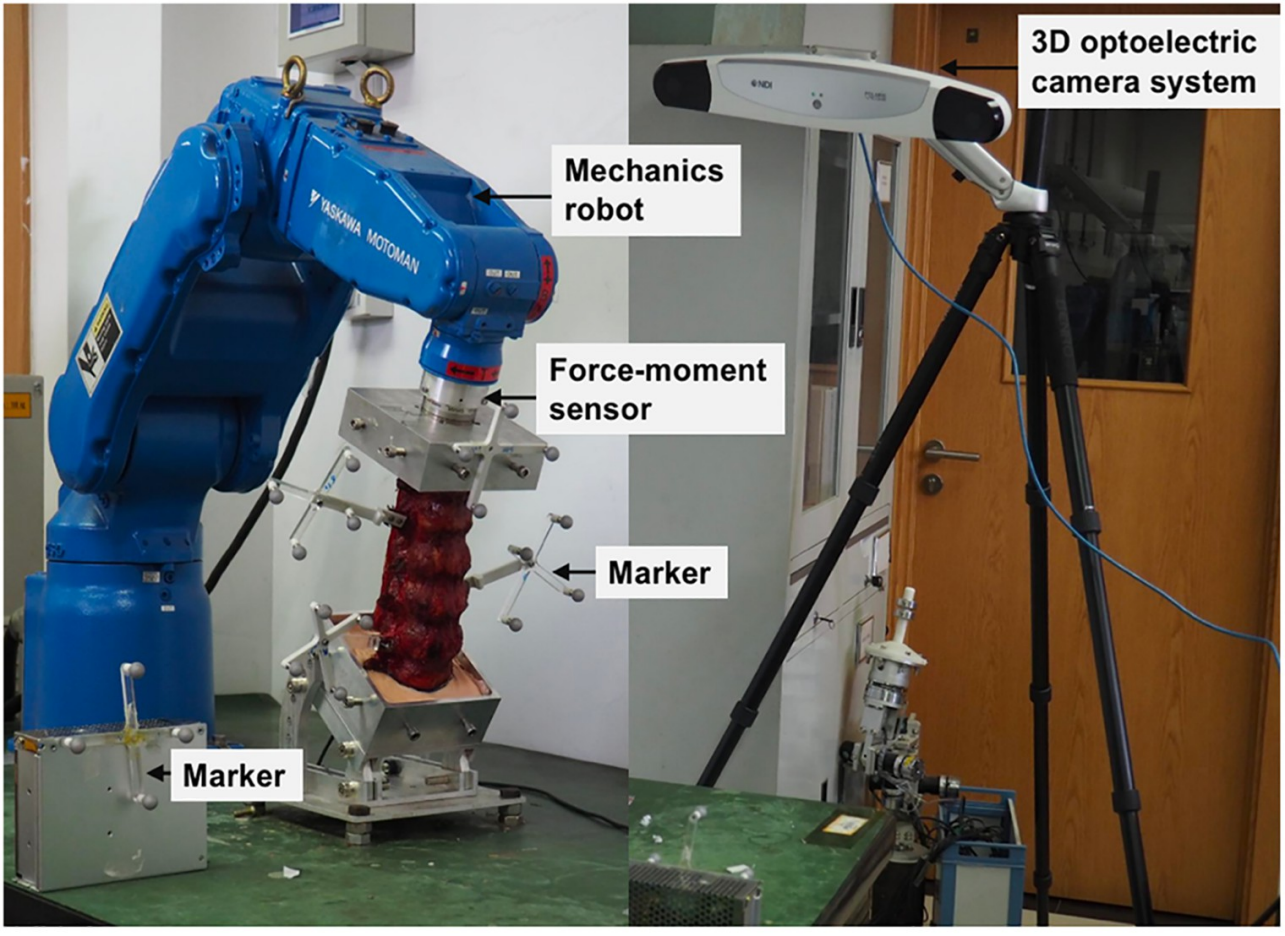
Image of the robotic testing device with a specimen embedded.

A robotic testing device was performed in this study, as shown in Fig 2, which has been used to measure the force-displacement behavior of lumbar segments in previous literature [17]. The upper vertebra L1 was connected to the six degrees of freedom robot (NX100MH6, Kabushiki-gaisha Yasukawa Denki, Kitakyushu, Japan), while the caudal vertebra S1 was embedded and fixed to the base frame. A force-moment sensor (Gamma, ATI Industrial Automation, Ontario, Canada) attached to the robotic system was used to record the applied forces and moments, and then provide feedback. The ROM was captured by recording the position of a set of markers based on a 3D optelectric camera system (Optotrak Certus, Northern Digital Inc, Waterloo, Canada). The center of the S1 and principal directions were determined by using the 3D spacial coordinate system of the camera system. In this study, five markers were fixed on the L1, L3, L4, L5 and the base.

A pure load control protocol was applied at a constant loading rate of 1.0°/s [16, 18]. In the testing, the specimen was tested under six pure moment cases: 7.5 Nm flexion, extension, right/left lateral bending and 5 Nm left/ right axial rotation. The robotic system determined the optimal loading path for each loading case from 0% to 100% of the magnitude of the target loading (7.5/5 Nm) in 10% increments. The specimen was subjected to 4.5 loading cycles. The first 1.5 was performed as pre-cycles to minimize the effect of viscoelastic response [16], and the data of the last 3 cycles were used for the following analysis.

### Construction of a based model

**Fig 3:**
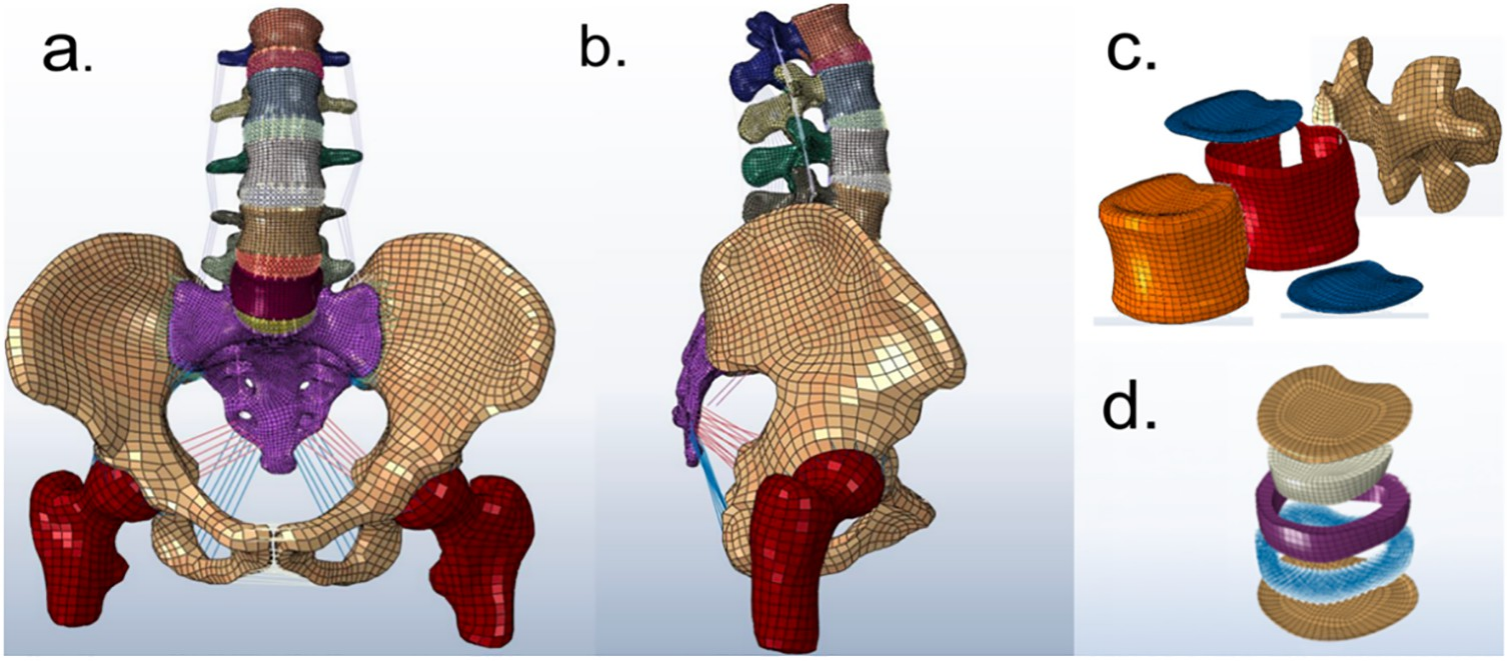
(a) Front and (b) lateral views of the FE model of the lumbar-pelvis; (b) Schematic of (c) the annulus fibrosis and (d) vertebra components.

A based lumbar-pelvis model (L1-S1-pelvis) was reconstructed based on the CT images of the human donor (Fig 3a–3c). The geometric structure was built by using Mimics software (Materialise, Belgium) and Geomagic Studio (Geomagic, America). Solidworks software (Dassault Systemes, France) was further refined the geometric modeling of the IVD. The IVD consisted of the nucleus pulposus (NP), the annulus fibrous (AF) and endplates (Fig 3d). The annulus fibrous was divided into seven layers, including the matrix and fibrous layers. A single fibrous layer was constructed with two-family fibers in the crossing-patterned directions (Fig 3c). These absolute values of the fiber angles increased from the ventral section (24°) to the dorsal section (46°) [19, 20]. The bony components and the IVDs were meshed using first-order hexahedral hybrid solid elements (C3D8). The annulus collagen fibers and the ligaments, including anterior (ALL) and posterior longitudinal ligament (PLL), flava (FL), supraspinous (SSL), interspinous (ISL), capsular ligaments (FC), were represented by tension truss elements(T2D2). The facet joint surfaces were modeled using surface to surface contact element without friction. The FE model approximately included 132,804 elements, 151,603 nodes and 451,200 degrees of freedom.

The material properties of the model are shown in Table 1 [20–22]. The fluid-like behavior of the NP and annulus matrix were assumed as nearly incompressible hyperelastic materials described by Mooney-Rivilin constitutive law. The tensile stress-strain of the collagen fibers was described by a non-linear function [23]. The facet joint surfaces were assumed as hard contact with a friction coefficient of 0.15. The facet cartilage layers with an initial clearance of 0.5 mm were described to be elastic isotropy (Young modulus of 35 MPa) [24].

**Table 1:** Material properties of the model.

Structure	Young’s modulus (MPa)	Poisson’s ratio
**Vertebrae**		
Cortical bone	E_x_ = 11,300; E_y_ = 11,30; E_z_ = 22,000; G_x_ = 3,800; G_y_ = 5,400G_z_ = 5,400	ν_xy_ = 0.484; ν_xz_ = 0.203; ν_yz_ = 0.203
Cancellous bone	E_x_ = 140; E_y_ = 140; E_z_ = 200; G_x_ = 48.3; G_y_ = 48.3; G_z_ = 48.3	ν_xy_ = 0.45; ν_xz_ = 0.315; ν_yz_ = 0.315
Posterior elements	3500	0.250
**Pelvis-Femur**		
Cortical bone	15000	0.30
Cancellous bone	100	0.20
**Disc**		
Nucleus pulposus	Hyperelastic, Mooney-Rivlin: C^10^ = 0.18, C^01^ = 0.045	
Annulus matrix	Hyperelastic, Mooney-Rivlin: C^10^ = 0.12, C^01^ = 0.03	
Fiber	Shirazi-adl’s stress-strain curve	
Endplate	3000	0.25
**Ligaments**		
ALL	7.8(< 12.0%), 20.0(> 12.0%)	0.40
PLL	10.0(< 11.0%), 20.0(> 11.0%)	0.30
SSL	8.0(< 20.0%), 15.0(> 20.0%)	0.30
ISL	10.0(< 14.0%), 11.6(>14.0%)	0.30
LF	15.8(< 6.2%), 19.5(> 6.2%)	0.30
TL	10.0(< 18.0%), 58.4(> 18.0%)	0.30
CL	7.5(< 25.0%), 32.9(> 25.0%)	0.30
ASL	125(<2.5%), 175(>5%),325(>10%),316(>15%)	0.30
IPSL	43(<2.5%), 61(>5%),113(>10%),110(>15%)	0.30
OPSL	150(<2.5%),211(>5%),391(>10%),381(>15%)	0.30
IL	40(<2.5%), 57(>5%),105(>10%),102(>15%)	0.30
SPL	304(<2.5%),428(>5%),792(>10%),771(>15%)	0.30
STL	326(<2.5%),458(>5%),848(>10%),826(>15%)	0.30

ALL: anterior longitudinal ligament; PLL: posterior longitudinal ligament; SSL: supraspinal ligament; ISL: interspinous ligament; LF: ligamentum flavum; TL: transverse ligaments; CL: capsular ligament; ASL:anterior sacroiliac ligament; IPSL: inner posterior sacroiliac ligament; OPSL: outer posterior sacroiliac ligament; IL:Intrerosseous ligament; SPL: Sacrospinous ligament; STL: Sacrotuberous ligament.

### Mesh convergence study

In the present study, linear hexahedron mesh and eight nodes quadratic tetra hedral (C3D8) elements type was considered for cortical bone, cancellous bone and posterior element. The annulus collagen fibers and the ligaments were represented by tension truss elements(T2D2) [25, 26]. The FE model approximately included 132,804 elements, 151,603 nodes and 451,200 degrees of freedom. A mesh convergence test was conducted to find the suitable mesh resolution for the FE model to confirm the accurateness of the simulation. The mesh density was found to produce well-converged results with element edge lengths of approximately 1–1.5 mm based on our previously published model [24, 27, 28]. Mesh convergence results showed less than a 5% difference in ROMs and disc loads when the number of solid elements was doubled in the model.

### Construction of four type sagittal models

To classify the four normal Roussouly’s sagittal spinopelvic morphotypes of the Chinese population, anteroposterior and lateral radiographs of 162 adults in a standardized standing posture were taken according to the previous retrospective observational study scheme [4, 6]. Subjects were divided into four Roussouly’s types based on the lumbar-pelvic radiographic parameters, including pelvic parameters (PI, PT and SS), lumbar parameters (lumbar lordosis (LL), Apex, upper arc, title angle) and the number of vertebrae in the lordosis (NVL), as shown in Table 2.

**Table 2:** Spino-pelvic parameters of Roussouly’s sagittal spino-pelvic morphotypes from a group of 160 subjects.

	Type 1	Type 2	Type 3	Type 4
**Size**	27	47	63	23
**Sex**	1.27	1.44	1.08	1.56
**Age**	48.71±17.11	51.83±15.30	46.06±18.13	52.17±23.00
**PI**	38.40±8.64	43.29±7.60	54.14±6.48	62.88±10.30
**PT**	10.09±8.77	10.94±8.65	11.74±6.78	12.53±9.14
**SS**	28.31±6.27	32.35±3.98	42.40±3.10	50.35±3.33
**LL**	42.96±8.07	47.77±8.22	56.97±7.42	67.95±3.03
**Apex**	Upper L5	Base L4	Middle L4	Base L3
**Upper arc**	14.65±4.15	15.42±3.73	14.57±9.81	17.60±5.32
**Title angle**	-7.91±2.21	-4.76±1.75	-6.33±4.73	-3.0±5.07
**NVL**	4.20 ±0.86	5.13±0.48	4.76±0.70	5.28±0.42

PI: pelvic incidence; PT: pelvic tilt; SS: sacral slope; LL: lumbar lordosis; NVL: number of vertebra in the lordosis.

**Fig 4:**
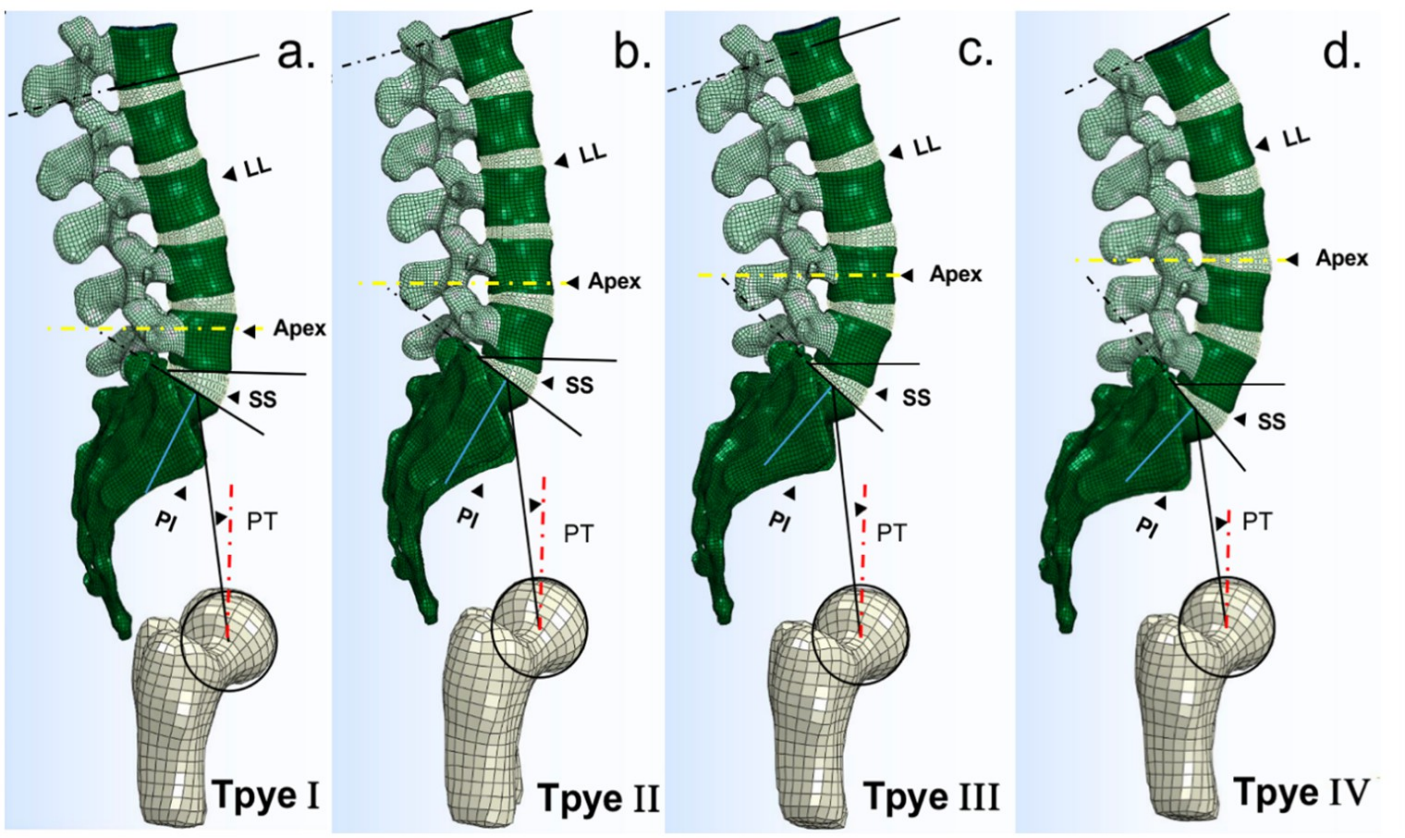
Schematic of finite element models of four Roussouly’s type. (a) Type 1, (b) Type 2, (c) Type 3, and (d) Type 4. PI: pelvic incidence; PT: pelvic tilt; SS: sacral slope; LL: lumbar lordosis.

The parametric FE models of Roussouly’s types were developed according to the based lumbar-pelvis model (L1-S1-pelvis) by using Solidworks software (Dassault Systemes, France) (Fig 4). Since the geometric parameters of the based model belonged to type 2, it served as type 2 model. Type 1, 3 and 4 models were reconstructed by adjusting the position of vertebral bodies in the based model to achieve targeted parameters of each type (Table 3). The geometric structures of the IVD and facet joints were modified based on the literature [18, 29, 30]. The lumbar-pelvic parameters of the four Roussouly’s type models were shown in Table 3.

**Table 3:** Spino-pelvic parameters of Roussouly’s type finite element models.

	Type 1	Type 2	Type 3	Type 4
**PI**	38.4	44.2	54.1	62.8
**PT**	10.1	11.4	11.7	12.5
**SS**	28.3	32.8	42.4	50.3
**LL**	42.9	48.2	56.9	67.9
**Apex**	Upper L5	Base L4	Middle L4	Base L3
**Upper arc**	14.6	15.4	14.5	17.6
**Title angle**	-7.9	-4.2	-6.3	-3.07
**NVL**	4.2	4.9	4.7	5.2

PI: pelvic incidence; PT: pelvic tilt; SS: sacral slope; LL: lumbar lordosis; NVL: number of vertebra in the lordosis.

### Boundary and loading conditions

In the FE models, the two femurs were rigidly fixed in all degrees of freedom. In the processing of modeling, we introduced a pilot node 50 mm above the disc center, and then rigidly coupled all nodes of the upper surface of the L1 IVD to the pilot node. A pure moment of 7.5 Nm was applied to the defined pilot node. The pure moment in the three main anatomical planes was assumed to simulate the physiological activities of flexion, extension, left-right lateral bending, and left-right axial rotation. The finite element program ABAQUS (SIMULIA Inc., Providence, Rhode Island, USA) was used for calculation.

### Data analysis

In the in vitro experiment, the moment-rotation curves under different loading conditions were calculated and compared with the measured curves simulated by the FE model to verify the validity of the model. Furthermore, the output data of four type models were measured and analyzed, including the intersegmental ROM, IDP and the maximal fiber of the disc in different loading scenarios. Cronbach’s α value was used to measure the reliability and validity of all the outcomes.

## Results

### Validation of the models

**Fig 5:**
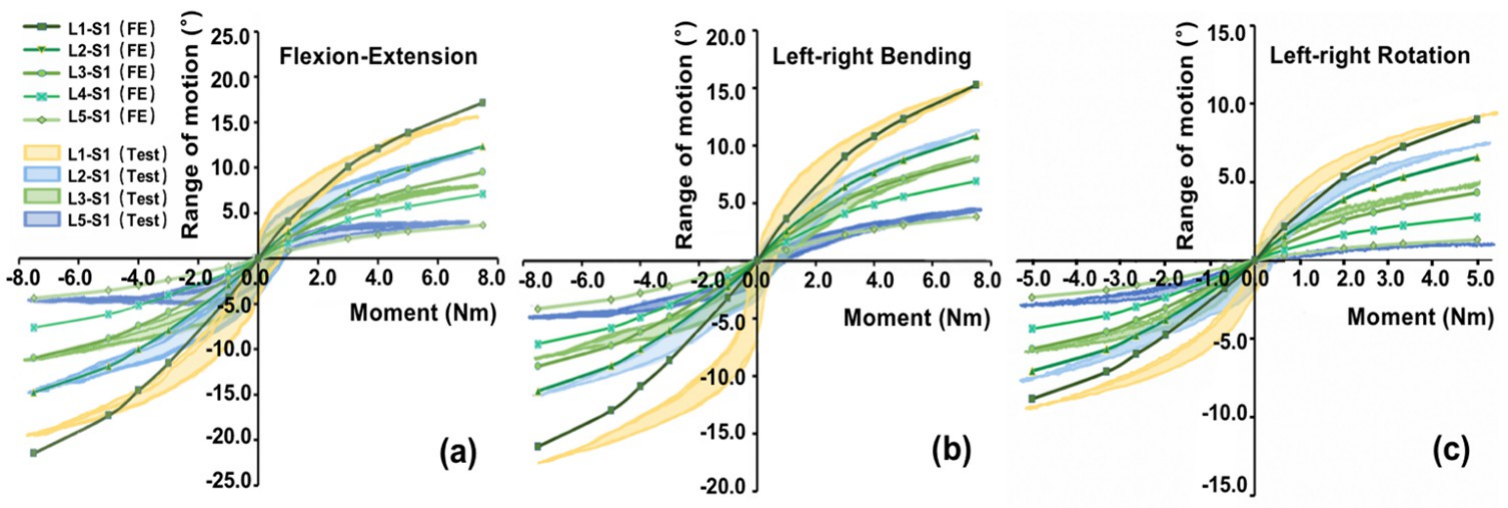
Comparison of the moment-rotation behaviors of the based lumbar-pelvis model with the in vitro experiments under different loads cases.

The moment-rotation behaviors of different segments calculated by the based lumbar-pelvis model had good agreement with those recorded hysteresis curves in the in-vitro experiments under different loading cases, as shown in Fig 5. The overall average errors were ~6%—~18% between FE modeling and in-vitro experiment results. Therefore, the based lumbar-pelvis model in this study was valid for further simulation of four Roussouly’s type models.

### Intervertebral rotations

**Fig 6:**
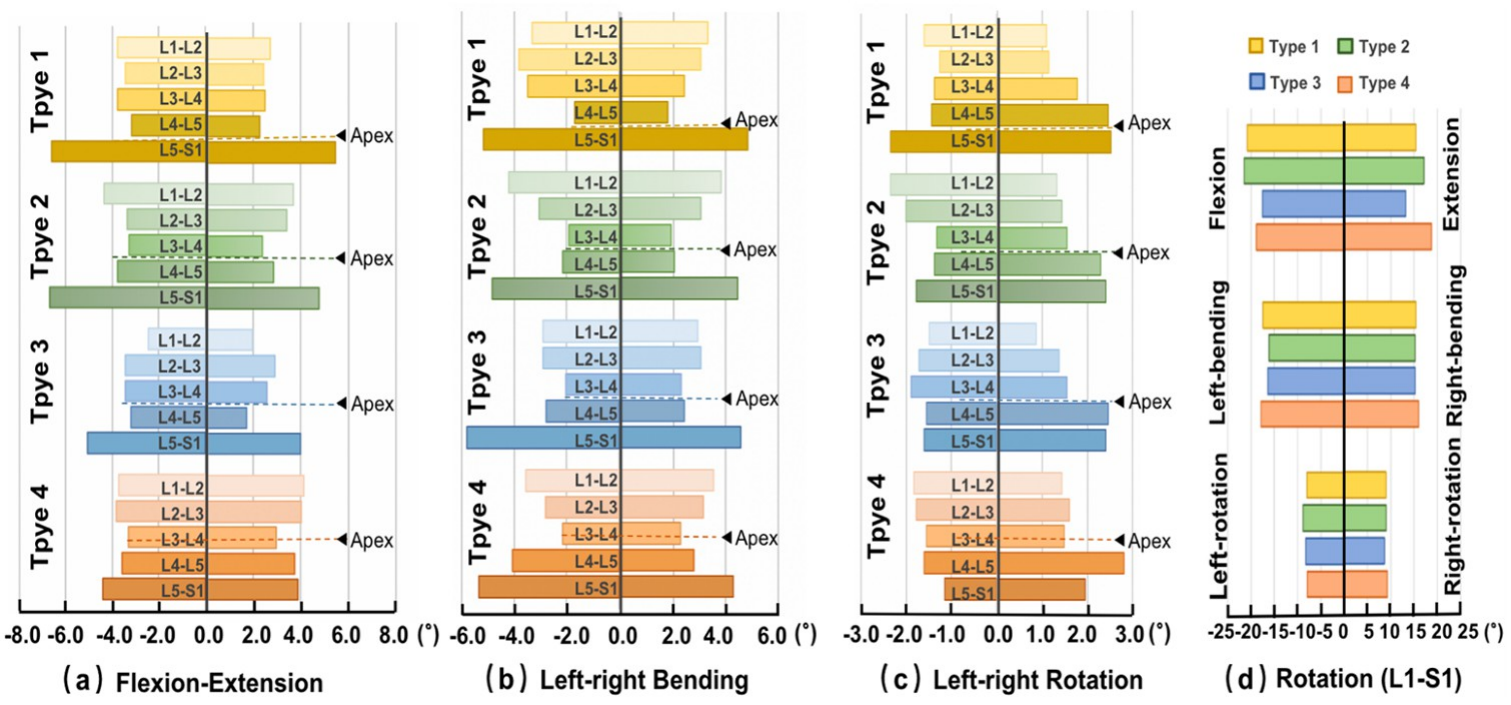
Rotation (°) of each segment of four Roussouly type FE models (a) in flexion-extension (7Nm), (b) lateral bending (7Nm), (c) axial rotation (5Nm) and (d) the L1-S2 segments in the six loading conditions.

The distribution of the intervertebral rotation for L1-L2, L2-L3, L3-L4, L4-L5, and L5-S1 segments was ranged in the four type models under different loading conditions (Fig 6). Under the flexion-extension moment (Fig 6a), the maximum ROM in all four type models occurred at the L5-S1 segments. The values were 6.62° in Type 1 model, 6.71° in type 2 model, 5.08° in type 3 model and 4.42° in type 4 model in flexion, respectively. The minimum ROM in type 1 (2.29° in extension) and type 3 models (1.75° in extension) appeared at L4-L5 segments. For type 2 and type 4 models, the minimum ROM at L3-L4 segments was 2.32°and 2.95° in extension, respectively. Under the lateral bending moment (Fig 6b), the maximum ROM was also at the L5-S1 segment, and the values were 5.17° in type 1 model, 4.83° in type 2 model, 5.80° in type 3 model, and 5.33° in type 4 model, respectively. The minimum was at L5-S1 segments in type 1 model of 1.68° and type 3 model of 2.24°, and at L3-L4 segments in type 2 model of 1.91° and type 4 model of 2.16°, respectively. Under the axial rotation moment (Fig 6c), the distribution of ROM varied between the different segments (Fig 6d). The Cronbach’s α of maximum ROM ranged from 0.87 to 0.98.

### Intradiscal pressures

**Fig 7:**
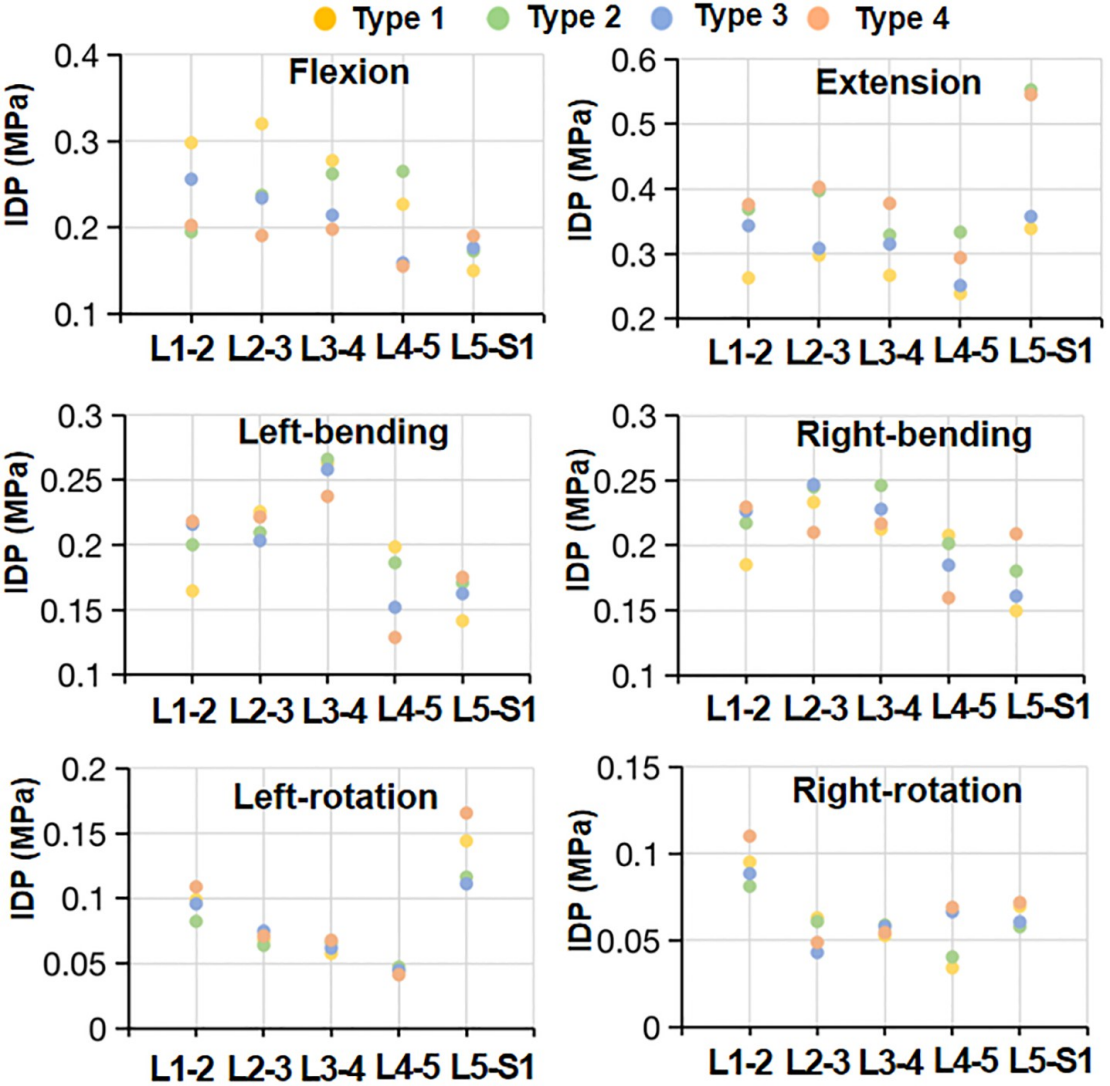
Scatter plots of intradiscal pressure (IDP) of the lumbar-sacral segments in the four sagittal type models for all six load cases.

In flexion, the IDPs (0.281–0.322MPa) at the L1-4 level of type1 model were larger than those (0.191–0.212MPa) of the other three models (Fig 7a). At the L4-5 level, the values of 0.227 Mpa in type1 model and 0.265 Mpa in type 2 model were higher than those of 0.159 Mpa in type 3 model and 0.156 Mpa in type 4 model. At the L5-S1 level, there was no significant difference in IDPs among the models. In extension, the IDPs of type 2 and type 4 models were generally higher than those of type 1 and type 3 models, especially at the L5-S1 level (Fig 7b). In lateral bending, the IDPs of the upper L1-4 discs were higher than those of the lower L4-S1 discs in all the models (Fig 7c and 7d). The values (0.131–0.27 MPa) of different L1-S1 segments showed little variation among the four type modes. In axial rotation, the distribution of IDPs along the L1-S1 segments in the four type models was the same, with a range of 0.035 ~ 0.110MPa (Fig 7e and 7f). The Cronbach’s α of the IDPs ranged from 0.85 to 0.96.

### Maximal matrix and fiber stress

**Fig 8:**
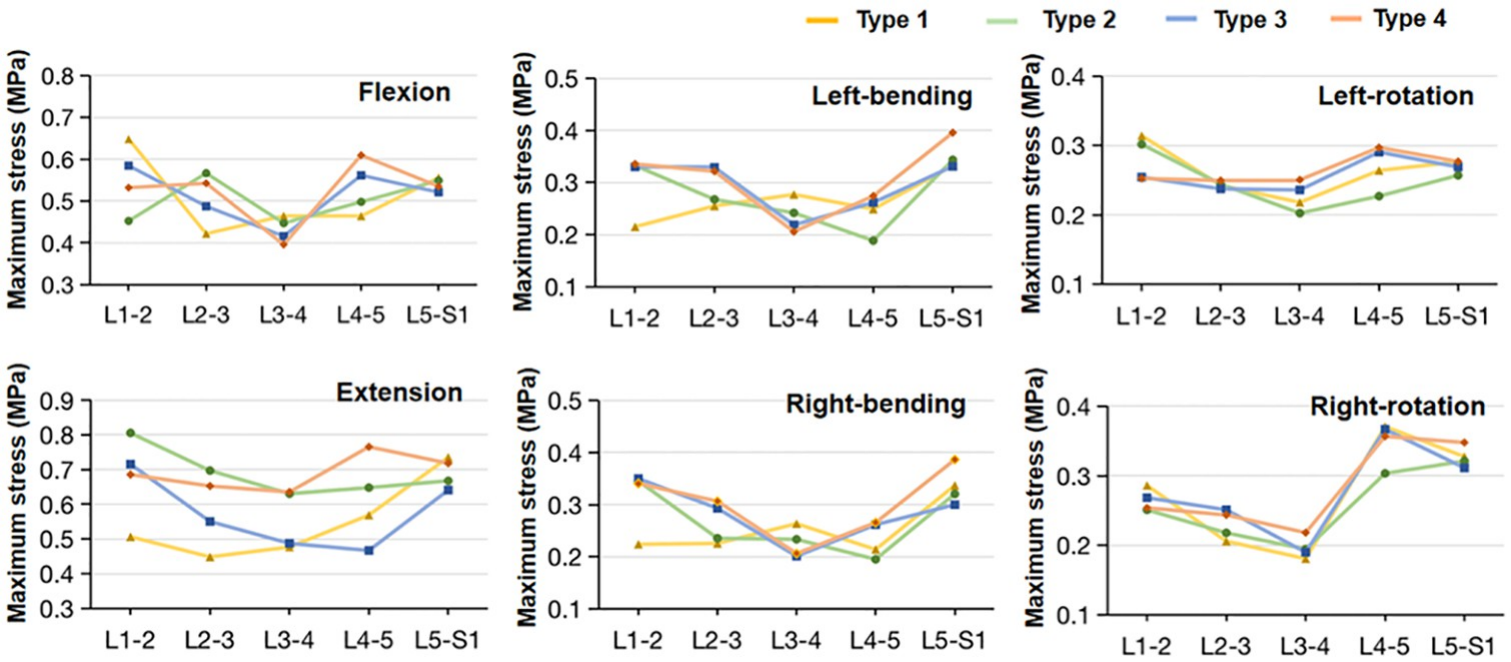
Maximum Von Mises stress of the annulus fibrous matrix of the lumbar-sacral segments in the four sagittal type models under all six loading conditions.

**Fig 9:**
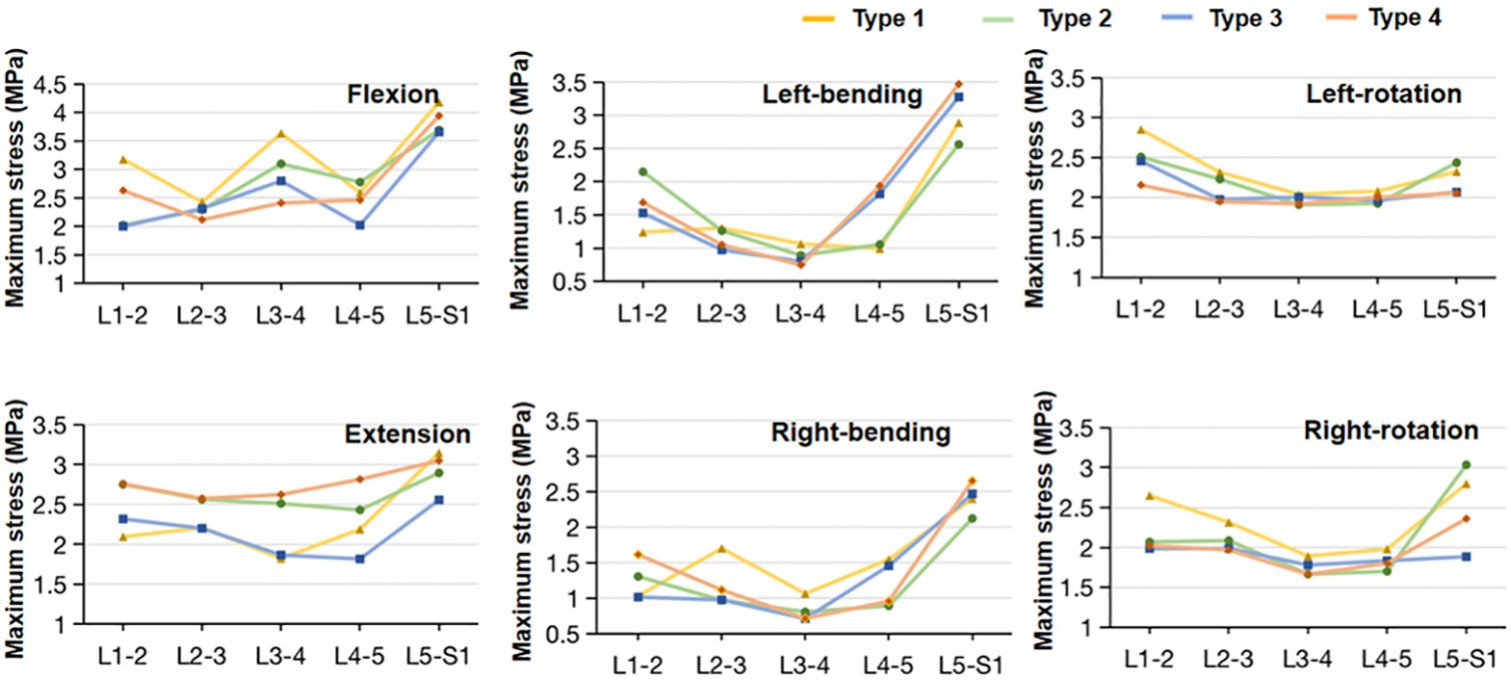
Maximum Von Mises stress of the annulus fibers of the lumbar-sacral segments in the four sagittal type models under all six loading conditions.

For flexion, the maximal matrix (0.648 MPa) and fiber stress (3.176 MPa) of type 1 model was larger than those of the other three models at the L1-2 level (Figs 8 and 9). At the L4-5 level, type 1 model (3.097 MPa) and type 2 model (3.623 MPa) had higher maximal fiber stress compared to type 3 model (2.321 MPa) and type 4 model (2.461 MPa), while the maximal matrix stress in type 1 model and type 2 model were smaller. At the L5-S1 level, the values of fiber stress in type 1 and type 2 models were slightly higher compared to type 2 and type 3 models, however, the difference of maximal matrix stress between the models was small. In extension, the maximal matrix and fiber stresses in type 2 and type 4 models were generally slightly larger than those in type 1 and type 3 models along with the L1-5 levels (Figs 8 and 9). Meanwhile, at the L5-S1 level, type 1 and type 4 models had higher matrix and fiber stresses compared to type 3 and type 4 models. For lateral bending, the largest maximal matrix and fiber stress was observed at the L5-S1 level in all the models (Figs 8 and 9). For axial rotation, the maximal matrix and fiber stresses along the L1-S1 levels had a similar tendency in all the models, while the values of maximal fiber stress in type 1 and type 2 models were generally slightly larger than those in type 3 and type 4 models (Figs 8 and 9). The Cronbach’s α of the maximal matrix and fiber stress ranged from 0.89 to 0.97.

## Discussion

In the past decades, sagittal alignment is a primordial factor in implementing and predicting spinal disorders and accurate surgical strategies. The classical Roussouly’s types (1–4) include the most common spinal types that have given reliable and clinical value to initially describe the sagittal spine alignment of a large asymptomatic cohort of the adult. The present study attempted to investigate the kinetic and mechanical characteristics of the four classical types of the spine according to the sagittal spinopelvic parameters of the Chinese population. The parametric FE models of Roussouly’s type (1–4) were developed with the based lumbar-pelvis model (L1-S1-pelvis) by adjusting the position of the vertebral structure to achieve all the average parameters for each Roussouly’s type (Table 3). The data showed that the sagittal alignment of Roussouly’s type models was associated with different kinetic and biomechanical responses, such as ROMs, IDPs, matrix and fiber stress, under the various loading conditions.

Numerous studies have proved the effect of the sagittal alignment on the biomechanical adaptation and compensation of the spine to obtain an economic physiological position. The sagittal configuration of four Roussouly’s type models had a strong effect on the biomechanical responses under sagittal loading conditions (in flexion and extension), a moderate effect under the lateral bending, but a weak effect under the axial rotation.

The effect of the sagittal configuration of four Roussouly’s type models on the biomechanical responses was strong, moderate and weak under sagittal loading conditions (in flexion and extension), the lateral bending and the axial rotation, respectively. The apex of the lumbar lordosis is a critical position where the segment has the lowest ROM in all the models under the sagittal and lateral bending loading conditions. That explained that the specific anatomic turning point was likely to determine the center of stability of the lumbar spine in relative movement. Sebaaly et.al indicated that restoring the sagittal apex of the lumbar lordosis had a positive effect in decreasing the incidence of proximal junctional kyphosis from 13.5% to 41.4% in cases [31]. In flexion-extension, type 3 and 4 models with a good lordosis shape had a more uniform rotation distribution at each motor function segment, while type 1 and 2 models with a straighter spine had a larger proportion of rotation at the L5-S1 level. Ferrero et al. [13] found that patients with low PT had a higher degree of disability before and after surgical treatment of adult spinal deformity [32]. Therefore, the well-marched lordotic type 3 lumbar had greater stability than those with less or more lordosis (e.g., types 1 and 4), while the more vertical type 2 lumbar had poor ability to control the balance under the sagittal bending loading conditions.

In summary, mechanical degeneration and disorders of the spine are not fully understood yet, but morphological sagittal plane parameters play an important role. We suggested that the L5-S1 segment in type 1 and 2 models accounted for a larger proportion of rotation, while the L4-5 segment had higher IDPs and matrix and fiber stresses in flexion. Coherently with previous investigations, Roussouly et al. and Adams et al. proposed that the high disc stress in type 1 and 2 patients tended to lead to early disc degeneration and herniation [4, 33]. Barrey et al. showed that young subjects with a flat back had a higher occurrence of disc herniation compared to those with marked lumbar lordosis [34]. Many authors have commented that vertical sacrum and less lumbar lordosis statistically have a higher risk to cause chronic low back pain [4, 35, 36]. When lumbar lordosis is relatively hypolordotic (type 4), this anatomical structure allowed for a larger range of motion of the spine, and had larger IDPs and matrix and fiber stresses at the L5-S1 segment in extension. The hypothesis was proposed that increasing lordosis of type 4 lumbar was prone to a higher risk of spinal spondylolisthesis and facet arthritis [14, 36]. The weakness of the posterior arch can lead to rupture (pedicle spondylolysis) or loosening (degenerative spondylolisthesis).

This study has several limitations. First, our model was reconstructed using the geometry of a single IVD extracted from CT images. Due to the complexity of Rousouly’s classification, the geometric parameters of the spine were not parameterized in this study. Future studies should further incorporate the statistical shape modeling of the spine into Roussouly’s classification. Second, the model was rebuilt based on the data of only Asian subjects, while morphological differences with Caucasian or African populations were not considered in this study. Third, the structural and material properties of our model were assumed as the average of the healthy human spine. The actual structure and materials of the spine (including degenerative discs, degenerative disc stents) were not optimized/simulated for comparison. Future studies should analyze the development of biomechanical responses of the four models at different degenerative stages. Finally, most muscles modeled as pure forces should be included and studied in the follow-up research. Despite these limitations, computer simulations can provide insights into the intrinsic biomechanics of normal spine-pelvis balance and a better understanding of how morphologic arrangements influence the evolution and degeneration of spinal diseases.

## Conclusion

This study showed that different Roussouly sagittal alignment morphotypes have various kinetic and mechanical characteristics under simulated physiological loading conditions. The apex of the lumbar lordosis is a critical position in the range of motion of the lumbar. Type 3 model had great stability in both motion and load. A straighter spine (type 1 and 2) had poor balance due to a larger proportion of rotation at the L5-S1 level. What’s more, type 1 and 2 models had higher intradiscal pressures. Type 4 model showed larger intradiscal pressures and matrix and fiber stresses at the L5-S1 segment. The findings could help improve our understanding of intrinsic biomechanics of the lumbar spine with different Roussouly’s type sagittal alignments.

## References

[1] Galbusera F, Wilke HJ, Brayda-Bruno M, Costa F, Fornari M. Influence of sagittal balance on spinal lumbar loads: A numerical approach. Clin Biomech. 2013;28: 370–377. doi: 10.1016/j.clinbiomech.2013.02.006 23489477

[2] Skoyles JR. Human balance, the evolution of bipedalism and dysequilibrium syndrome. Med Hypotheses. 2006. doi: 10.1016/j.mehy.2006.01.042 16530977

[3] Poincar R, Several I, Duval G, Poincar R, Duval-Beaupere G, Schmidt C, et al: A Barycentremetic Study of the Sagittal Shape of Spine and Pelvis: The Conditions Required for an Economic Standing Position. Annals of Biomedical Engineering, Volume 20, pp 451–462, 1992. Ann Biomed Eng. 1992;20: 451–462. doi: 10.1007/BF02368136 1510296

[4] Roussouly P, Pinheiro-Franco JL. Biomechanical analysis of the spino-pelvic organization and adaptation in pathology. Eur Spine J. 2011;20 Suppl 5: 609–618. doi: 10.1007/s00586-011-1928-x 21809016 PMC3175914

[5] Roussouly P, Nnadi C. Sagittal plane deformity: An overview of interpretation and management. European Spine Journal. 2010. pp. 1824–1836. doi: 10.1007/s00586-010-1476-9 20567858 PMC2989270

[6] Roussouly P, Gollogly S, Berthonnaud E, Dimnet J. Classification of the normal variation in the sagittal alignment of the human lumbar spine and pelvis in the standing position. Spine (Phila Pa 1976). 2005;30: 346–353. doi: 10.1097/01.brs.0000152379.54463.65 15682018

[7] Wang HJ, Giambini H, Zhang WJ, Ye GH, Zhao C, An KN, et al. A modified sagittal spine postural classification and its relationship to deformities and spinal mobility in a Chinese osteoporotic population. PLoS One. 2012;7. doi: 10.1371/journal.pone.0038560 22693647 PMC3367929

[8] Yu M, Zhao WK, Li M, Wang SB, Sun Y, Jiang L, et al. Analysis of cervical and global spine alignment under Roussouly sagittal classification in Chinese cervical spondylotic patients and asymptomatic subjects. Eur Spine J. 2015;24: 1265–1273. doi: 10.1007/s00586-015-3832-2 25805575

[9] During J, Goudfrooij H, Keessen W, Beeker TW, Crowe A. Toward standards for posture: Postural characteristics of the lower back system in normal and pathologic conditions. Spine. 1985. pp. 83–87. doi: 10.1097/00007632-198501000-00013 3157224

[10] Sebaaly A, Gehrchen M, Silvestre C, Kharrat K, Bari TJ, Kreichati G, et al. Mechanical complications in adult spinal deformity and the effect of restoring the spinal shapes according to the Roussouly classification: a multicentric study. Eur Spine J. 2020;29: 904–913. doi: 10.1007/s00586-019-06253-1 31875922

[11] Bari TJ, Hansen LV, Gehrchen M. Surgical correction of Adult Spinal Deformity in accordance to the Roussouly classification: effect on postoperative mechanical complications. Spine Deform. 2020;8: 1027–1037. doi: 10.1007/s43390-020-00112-6 32279244

[12] Le Huec JC, Faundez A, Dominguez D, Hoffmeyer P, Aunoble S. Evidence showing the relationship between sagittal balance and clinical outcomes in surgical treatment of degenerative spinal diseases: a literature review. Int Orthop. 2015;39: 87–95. doi: 10.1007/s00264-014-2516-6 25192690

[13] Galbusera F, Brayda-Bruno M, Costa F, Wilke HJ. Numerical evaluation of the correlation between the normal variation in the sagittal alignment of the lumbar spine and the spinal loads. J Orthop Res. 2014;32: 537–544. doi: 10.1002/jor.22569 24375659

[14] Gohari S, Mozafari F, Moslemi N, Mouloodi S, Sharifi S, Rahmanpanah H, et al. Analytical solution of the electro-mechanical flexural coupling between piezoelectric actuators and flexible-spring boundary structure in smart composite plates. Arch Civ Mech Eng. 2021;21: 1–25.

[15] Gohari, Soheil, Sharifi, S., Vrcelj, Zora. A novel explicit solution for twisting control of smart laminated cantilever composite plates/beams using inclined piezoelectric actuators. Compos Struct. 2017.

[16] Wilke H-J, Wenger K, Claes L. Testing criteria for spinal implants. Euro Spine J. 1998. doi: 10.1007/s005860050045 9629939 PMC3611233

[17] Kong C, Lu S, Hai Y, Zang L. Biomechanical effect of interspinous dynamic stabilization adjacent to single-level fusion on range of motion of the transition segment and the adjacent segment. Clin Biomech. 2015;30: 355–359. doi: 10.1016/j.clinbiomech.2015.02.012 25779689

[18] Panjabi MM, Goel V, Oxland T, Takata K, Duranceau J, Krag M, et al. Human lumbar vertebrae: Quantitative three-dimensional anatomy. Spine (Phila Pa 1976). 1992. doi: 10.1097/00007632-199203000-00010 1566168

[19] Holzapfel GA, Schulze-Bauer CAJ, Feigl G, Regitnig P. Single lamellar mechanics of the human lumbar anulus fibrosus. Biomech Model Mechanobiol. 2005;3: 125–140. doi: 10.1007/s10237-004-0053-8 15778871

[20] Eberlein R, Holzapfel GA, Schulze-Bauer CAJ. An anisotropic model for annulus tissue and enhanced finite element analyses of intact lumbar disc bodies. Comput Methods Biomech Biomed Engin. 2001. doi: 10.1080/10255840108908005

[21] Lu YM, Hutton WC, Gharpuray VM. Can variations in intervertebral disc height affect the mechanical function of the disc? Spine (Phila Pa 1976). 1996;21: 2208–2217. doi: 10.1097/00007632-199610010-00006 8902964

[22] Sharma M, Langrana NA, Rodriguez J. Role of ligaments and facets in lumbar spinal stability. Spine (Phila Pa 1976). 1995;20: 887–900. doi: 10.1097/00007632-199504150-00003 7644953

[23] Shirazi-Adl A, Ahmed AM, Shrivastava SC. A finite element study of a lumbar motion segment subjected to pure sagittal plane moments. J Biomech. 1986;19: 331–350. doi: 10.1016/0021-9290(86)90009-6 3711133

[24] Wang W, Pei B, Pei Y, Shi Z, Kong C, Wu X, et al. Biomechanical effects of posterior pedicle fixation techniques on the adjacent segment for the treatment of thoracolumbar burst fractures: a biomechanical analysis. Comput Methods Biomech Biomed Engin. 2019;22. doi: 10.1080/10255842.2019.1631286 31225742

[25] Gohari Soheil, Sharifi, S, Vrcelj, Zora. New explicit solution for static shape control of smart laminated cantilever piezo-composite-hybrid plates/beams under thermo-electro-mechanical loads using piezoelectric actuators. Compos Struct. 2016.

[26] Katsamakas AA, Papanikolaou VK, Thermou GE. A FEM-based model to study the behavior of SRG-strengthened R/C beams. Compos Struct. 2021.

[27] Zhou C, Willing R. Sensitivities of lumbar segmental kinematics and functional tissue loads in sagittal bending to design parameters of a ball-in-socket total disc arthroplasty prosthesis. Comput Methods Biomech Biomed Engin. 2020;23: 536–547. doi: 10.1080/10255842.2020.1745783 32251611

[28] Gohari S, Sharifi S, Burvill C, Mouloodi S, Izadifar M, Thissen P. Localized failure analysis of internally pressurized laminated ellipsoidal woven GFRP composite domes: Analytical, numerical, and experimental studies. Arch Civ Mech Eng. 2019;19: 1235–1250. doi: 10.1016/j.acme.2019.06.009

[29] O’Connell GD, Vresilovic EJ, Elliott DM. Comparison of animals used in disc research to human lumbar disc geometry. Spine (Phila Pa 1976). 2007. doi: 10.1097/01.brs.0000253961.40910.c1 17268264

[30] Been E, Li L, Hunter DJ, Kalichman L. Geometry of the vertebral bodies and the intervertebral discs in lumbar segments adjacent to spondylolysis and spondylolisthesis: Pilot study. Eur Spine J. 2011. doi: 10.1007/s00586-010-1660-y 21181481 PMC3176698

[31] Sebaaly A, Riouallon G, Obeid I, Grobost P, Rizkallah M, Laouissat F, et al. Proximal junctional kyphosis in adult scoliosis: comparison of four radiological predictor models. Eur Spine J. 2018. doi: 10.1007/s00586-017-5172-x 28597300

[32] Ferrero E, Vira S, Ames CP, Kebaish K, Obeid I, O’Brien MF, et al. Analysis of an unexplored group of sagittal deformity patients: low pelvic tilt despite positive sagittal malalignment. Eur Spine J. 2016;25: 3568–3576. doi: 10.1007/s00586-015-4048-1 26026474

[33] Adams MA, Roughley PJ. What is intervertebral disc degeneration, and what causes it? Spine. 2006. pp. 2151–2161. doi: 10.1097/01.brs.0000231761.73859.2c 16915105

[34] Barrey C, Jund J, Noseda O, Roussouly P. Sagittal balance of the pelvis-spine complex and lumbar degenerative diseases. A comparative study about 85 cases. Eur Spine J. 2007;16: 1459–1467. doi: 10.1007/s00586-006-0294-6 17211522 PMC2200735

[35] Hey HWD, Wong GC, Chan CX, Lau LL, Kumar N, Thambiah JS, et al. Reproducibility of sagittal radiographic parameters in adolescent idiopathic scoliosis—a guide to reference values using serial imaging. Spine J. 2017;17: 830–836. doi: 10.1016/j.spinee.2017.01.001 28065817

[36] Sebaaly A, Grobost P, Mallam L, Roussouly P. Description of the sagittal alignment of the degenerative human spine. Eur Spine J. 2018;27: 489–496. doi: 10.1007/s00586-017-5404-0 29177554

